# Critically Ill *vs*. Non-Critically Ill Patients With COVID-19 Pneumonia: Clinical Features, Laboratory Findings, and Prediction

**DOI:** 10.3389/fcimb.2021.550456

**Published:** 2021-07-13

**Authors:** Wandong Hong, Qin Chen, Songzan Qian, Zarrin Basharat, Vincent Zimmer, Yumin Wang, Maddalena Zippi, Jingye Pan

**Affiliations:** ^1^ Department of Gastroenterology and Hepatology, The First Affiliated Hospital of Wenzhou Medical University, Wenzhou, China; ^2^ Department of Intensive Care Unit, The First Affiliated Hospital of Wenzhou Medical University, Wenzhou, China; ^3^ Jamil-ur-Rahman Center for Genome Research, Dr. Panjwani Centre for Molecular Medicine and Drug Research, International Center for Chemical and Biological Sciences, University of Karachi-75270, Karachi, Pakistan; ^4^ Department of Medicine II, Saarland University Medical Center, Saarland University, Homburg, Germany; ^5^ Department of Medicine, Marienhausklinik St. Josef Kohlhof, Neunkirchen, Germany; ^6^ Department of Laboratory Medicine, The First Affiliated Hospital of Wenzhou Medical University, Wenzhou, China; ^7^ Unit of Gastroenterology and Digestive Endoscopy, Sandro Pertini Hospital, Rome, Italy; ^8^ Intensive Care Unit, The First Affiliated Hospital of Wenzhou Medical University, Wenzhou, China

**Keywords:** COVID-19, infection, pneumonia, severity, critically ill, predictor

## Abstract

**Objectives:**

The objective of this study was to investigate the clinical features and laboratory findings of patients with and without critical COVID-19 pneumonia and identify predictors for the critical form of the disease.

**Methods:**

Demographic, clinical, and laboratory data of 63 COVID-19 pneumonia patients were retrospectively reviewed. Laboratory parameters were also collected within 3–5 days, 7–9 days, and 11–14 days of hospitalization. Outcomes were followed up until March 12, 2020.

**Results:**

Twenty-two patients developed critically ill pneumonia; one of them died. Upon admission, older patients with critical illness were more likely to report cough and dyspnoea with higher respiration rates and had a greater possibility of abnormal laboratory parameters than patients without critical illness. When compared with the non-critically ill patients, patients with serious illness had a lower discharge rate and longer hospital stays, with a trend towards higher mortality. The interleukin-6 level in patients upon hospital admission was important in predicting disease severity and was associated with the length of hospitalization.

**Conclusions:**

Many differences in clinical features and laboratory findings were observed between patients exhibiting non-critically ill and critically ill COVID-19 pneumonia. Non-critically ill COVID-19 pneumonia also needs aggressive treatments. Interleukin-6 was a superior predictor of disease severity.

## Highlights

1. The mortality rate in critically ill patients is low.
2. Different severity of diseases has different clinical and laboratory results.
3. Non-critically ill COVID-19 pneumonia also needs aggressive treatments.
4. Interleukin-6 is a good predictor of critically ill COVID-19 pneumonia.
5. Interleukin-6 is associated with length of hospitalization

## Introduction

Novel coronavirus (COVID-19) pneumonia is a newly recognized disease that has spread rapidly throughout China, originating from Wuhan (Hubei province) and expanding to other provinces within the country and around the world (Yang et al., 2020). The current novel coronavirus has surpassed Severe Acute Respiratory Syndrome (SARS) in terms of the number of recorded cases and deaths from the disease (Peeri et al., 2020). The clinical spectrum of COVID-19 pneumonia ranges from mild to critically ill (Yang et al., 2020). Yang et al. have reported on clinical courses and outcomes of critically ill patients with COVID-19 pneumonia in Wuhan, China (Yang et al., 2020). There has been no comparison of data between patients with and without severe COVID-19 pneumonia. In addition, critically ill patients had a high mortality rate of 61·5% and often had to be transferred to an intensive care unit (ICU). Therefore, it is important to recognize predictors of disease severity in the early phase of COVID-19 infection. This could help select patients who could benefit from close surveillance or aggressive interventions. Early case recognition and classification of disease severity improves clinical outcomes (Liao et al., 2020).

Therefore, our study aimed to investigate the clinical features of COVID-19 pneumonia in patients who were critically ill *vs.* those who were non-critically ill and identify possible predictors of disease severity.

## Materials and Methods

### Study Design, Subject Selection

We conducted a retrospective cohort study in the First Affiliated Hospital of Wenzhou Medical University in mainland China. All patients with confirmed COVID-19 pneumonia between January 29, 2020 and March 12, 2020 were eligible for inclusion in this study. A confirmed case of COVID-19 was defined as exhibiting a positive result on high-throughput sequencing or real-time reverse-transcriptase–polymerase chain reaction (RT-PCR) assay of nasal and pharyngeal swab specimens (Guan et al., 2020). Exclusion criteria were the unavailability of chest computed tomography (CT) scans.

### Definition of Severity

According to the China Guidelines for the Diagnosis and Treatment Plan of COVID-19 Infection (2020; Xu Y. H. et al., 2020), COVID-19 infections are classified into four types on admission: critically severe type with any of the following: respiratory failure needing mechanical ventilation, shock, or combination with different organ failure requiring admission into an intensive care unit (ICU); severe type with any of the following: respiratory distress with respiratory rate>30 times/minutes, oxygen saturation at rest <93%, or PaO2/FiO2 <300 mmHg; common with fever, respiratory symptoms, and imaging presentations of pneumonia; and, mild with slight clinical symptoms but no imaging presentations of pneumonia (2020).

For comparative analysis, we also defined the degree of severity of critically ill *vs.* non critically ill cases of COVID-19 according to a previous study (Kumar et al., 2009). Critically ill patients were defined as those admitted to the ICU and either required mechanical ventilation or had a fraction of inspired oxygen (FiO2) value of at least 60% or more during hospitalization (Kumar et al., 2009; Yang et al., 2020). The date of disease onset was defined as the day when the symptoms were first noticed (Wang et al., 2020).

### Data Collection, Follow up and Ethics

The epidemiological, clinical, laboratory, radiologic, and treatment and outcomes data during the course of hospitalization were obtained with data collection forms from electronic medical records. The date of disease onset was defined as the day when the symptoms were noticed (Wang et al., 2020). Fever was defined as any value over normal body temperature (>37.0°C) during the time from symptoms to admission. Also, body temperature was measured at admission as a sign of the disease. Chronic concomitant diseases, alcohol consumption and smoking were also recorded at the time of admission (Hong et al., 2017; Hong et al., 2020). Longitudinal data of laboratory parameters at different time points, i.e. within 24 h, 3–5 days, 7–9 days, and 11–14 days after admission, were collected and analyzed. Outcomes were followed up until March 12, 2020.

This study protocol was approved by the Ethics Committee of the First Affiliated Hospital of Wenzhou Medical University. It was performed according to the principles expressed in the Declaration of Helsinki, and informed consent was obtained from all the subjects.

### Statistical Analysis

The Shapiro-Wilk test was used to determine if the continuous data follow a normal distribution (Hong et al., 2020). Continuous values were expressed by mean ± SD or median and Inter Quartile Range (IQR) and compared using one-way analysis of variance (ANOVA) or the Kruskal-Wallis non-parametric test. Categorical values were described by count and proportions and compared by the χ2 test or Fisher’s exact test.

The area under the receiver operating characteristic (ROC) curve (AUC) was used to evaluate the performance of the predictors. A larger AUC value is indicative of greater diagnostic accuracy of a variable (Hong et al., 2011). An AUC above 0·8 indicates good diagnostic accuracy for a variable (Hong et al., 2017). The best cut-off point was set as the point where the number of false positives is as low as possible (specificity>95%) and is determined by selecting a threshold value at the point where the longest increase in the sensitivity of the slope declines (Hong et al., 2017). The sensitivity, specificity, negative predictive value, positive predictive value, positive likelihood ratio, negative likelihood ratio, and diagnostic accuracy were all calculated for the various corresponding cut-off values.

Pearson correlation and linear regression analysis were used to investigate the relationship between predictor and length of hospitalization. The variables with skewed distribution were log-transformed for correlation analysis when necessary. Differences were considered to be statistically significant if the two-tailed P value was less than 0.05.

## Results

### Clinical Characteristics

A total of 63 hospitalized patients confirmed to have COVID-19 pneumonia were enrolled in our study. Twenty-eight patients were imported cases who had traveled from Wuhan City (**Supplementary Figure 1**). None of the medical staff were infected. As shown in **Table 1**, the mean age of the pneumonia patients was 55·9 ± 15·3 (range: 17-92) years. Forty-one (65·1%) patients were men. The mean time from onset of symptoms to admission into our hospital was 6·9 ± 3·7 days. A few of the patients had a history of smoking (17·5%) and alcohol intake (16·0%). Twenty-seven (42·9%) patients had chronic concomitant diseases. The most common symptoms at the onset of the illness were fever (98·4%), cough (61·94%) and sputum (34·9%). However, 16 (25·4%) patients had normal body temperatures at admission. This means that the fever maybe not persist from onset to admission. Sixty-one (96·8%) of the patients had bilateral involvement of pneumonia in chest CT images (**Table 1**). The typical findings from chest CT images upon admission were local high-density patches, masses (**Supplementary Figure 2A**), and ground-glass opacity (**Supplementary Figure 2B**).

**Table 1:** Baseline characteristics of 63 patients infected with COVID-19 pneumonia on admission.

Characteristic	Total number (N=63)	Non- Critically ill (N=41)	Critically ill (N=22)	P-value
Median age, years (IQR)	56 ± 15	52·9 ± 14·9	61·5 ± 14·7	0·034
Male sex, N (%)	41 (65·1)	26 (63·4)	15 (68·2)	0·705
Time from symptoms to admission, days	6·9 ± 3·7	6·9 ± 4·0	6·8 ± 2·8	0·939
**Epidemic data**				0.847
Travelled from Wuhan, N (%)	25 (38·1)	16 (39·0)	9 (40·9)	
Contacted to case, N (%)	14 (22·2)	10 (24·4)	4 (18·2)	
Occult history, N (%)	24 (39·7)	15 (36·6)	9 (40·9)	
Median BMI	25·2 (22·3–26·9) (N=40)	24·5 (22.1–26.4) (N=28)	25·9 (24·5–29·0) (N=12)	0·165
Smoking, N (%)	11 (17·5)	6 (14·6)	5 (22·7)	0·420
Alcohol, N (%)	10 (16·0)	6 (14·6)	4 (18·2)	0·713
**Chronic concomitant diseases**, N (%)	27 (42·9)	15 (36·6)	12 (54·6)	0·170
Hypertension, N (%)	21 (33·3)	12 (29·3)	9 (40·9)	0·407
Diabetes mellitus, N (%)	9 (14·3)	4 (9·8)	5 (22·7)	0·256
Malignancy, N (%)	2 (3·2)	0 (0)	2 (9·1)	0·118
Cardiovascular, N (%)	2 (3·2)	2 (4·9)	0 (0)	0·538
Neurologic, N (%)	1 (1·6)	1 (2·4)	0 (0)	1·000
Pulmonary, N (%)	1 (1·6)	0 (0)	1 (4·5)	0·349
Hepatitis virus carrier, N (%)	1 (1·6)	1 (2·4)	0 (0)	1·000
**Symptoms**				
Fever, N (%)	62 (98·4)	40 (97·6)	22 (100)	1·000
Cough, N (%)	39 (61·9)	21 (51·2)	18 (81·8)	0·028
Sputum, N (%)	22 (34·9)	12 (29·3)	10 (45·5)	0·269
Dyspnoea, N (%)	17 (27·0)	4 (9·8)	13 (59·1)	<0·001
Chills, N (%)	15 (23·8)	12 (29·3)	3 (13·6)	0·222
Fatigue, N (%)	8 (12·7)	4 (9·8)	4 (18·2)	0·434
Sore throat, N (%)	7 (11·1)	6 (14·6)	1 (4·6)	0·405
Headache, N (%)	3 (4·8)	3 (7·3)	0 (0)	0·546
Myalgia, N (%)	4 (6·4)	2 (4·9)	2 (9·1)	0·606
Diarrhea, N (%)	3 (4·8)	2 (4·9)	1 (4·6)	1·000
**Signs**				
Distribution of temperature				0.128
<37·0, N (%)	16 (25·4)	14 (34·1)	2 (9·1)	
37·0-37·4, N (%)	17 (27·0)	9 (22·0)	8 (36·4)	
37·5-38·0, N (%)	15 (23·8)	10 (24·4)	5 (22·7)	
38·1-39, N (%)	14 (22·2)	7 (17·1)	7 (31·8)	
>39, N (%)	1 (1·6)	1 (2·4)	0 (0)	
Mean arterial pressure (IQR), mmHg	99·0 ± 11·5	98·6 ± 11·4	99·6 ± 11·9	0·741
Heart rate, bpm	87·1 ± 16·0	88·2 ± 16·7	84·9 ± 14·7	0·438
Respiratory rate	20 (20–23)	20 (20–20)	23·5 (20–28)	0·004
**Location of CT findings**				0·538
Unilateral pneumonia	2 (3·2)	2 (4·9)	0 (0)	
Bilateral pneumonia	61 (96·8)	39 (95·1)	22 (100)	

Data are shown either as the number of observations, percentage, or median and interquartile range.

**Figure 1:**
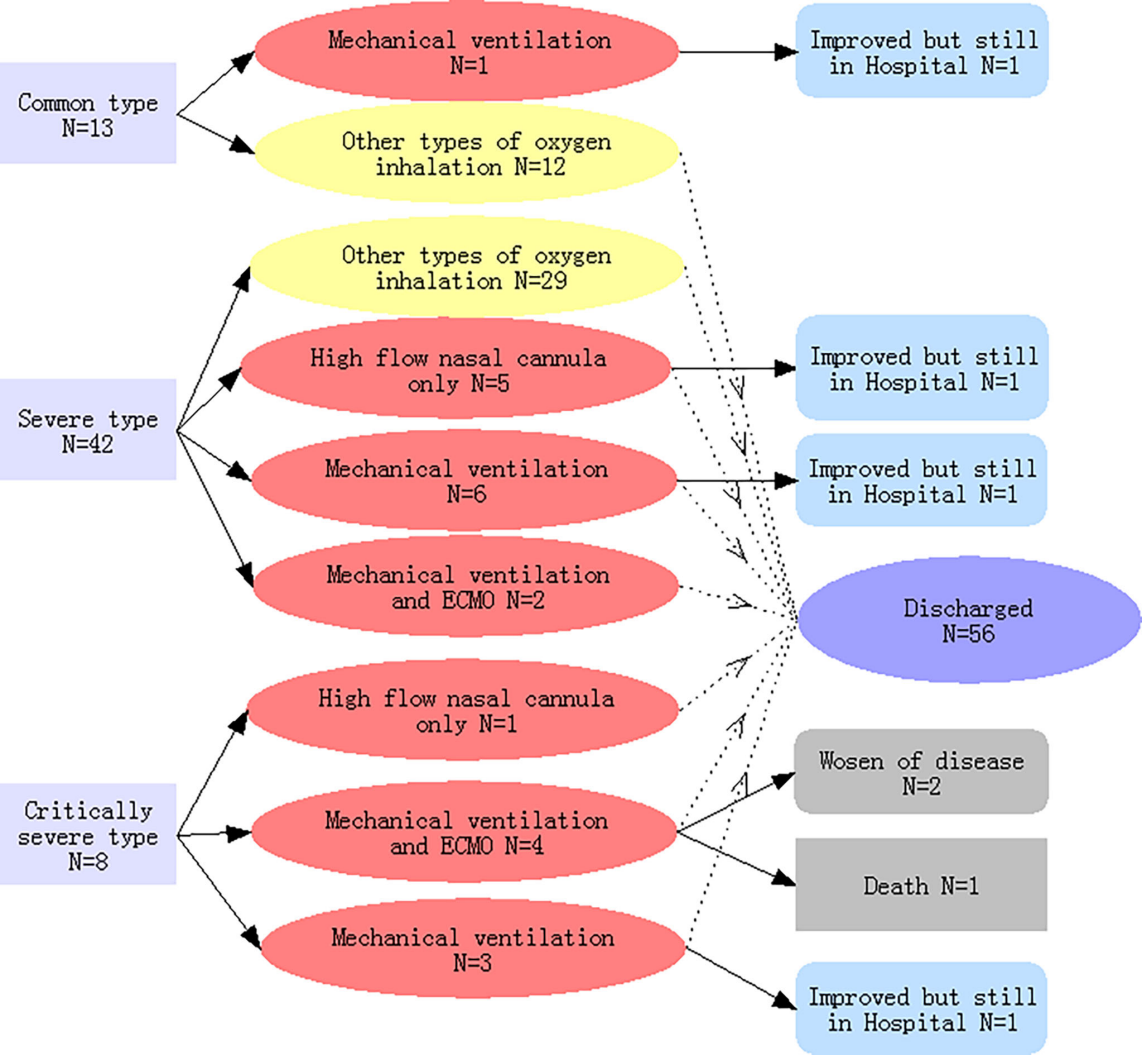
Classifications of the severity of COVID-19-related pneumonia on admission, types of oxygen inhalation, and clinical outcomes. Light yellow indicates COVID-19 pneumonia patients with non-critical illness; light red indicated patients with critical illness.

As shown in **Figure 1**, upon admission, there were 13, 42, and 8 patients with common type, severe type, and critically severe type pneumonia, respectively, in our study. One COVID-19 pneumonia patient from the common type group and 13 COVID-19 pneumonia patients from the severe type group progressed to the critically ill type during hospitalization. Therefore, of the 63 patients, 22 (34·9%) required high-flow nasal cannula at a later stage or higher-level oxygen support measures to correct hypoxemia during hospitalization and were classified as critically ill patients, while the remainder (41 patients) were recorded as non-critically ill patients.

As shown in **Table 1**, in comparison with the non-critically ill patients, patients with critical illness were generally older and were more likely to report cough and dyspnoea with higher respiratory rate frequency.

### Laboratory Findings

Upon admission, data regarding levels of D-dimer (full data available for 58 patients), B-type natriuretic peptide (full data available for 62 patients), and interleukin-6 (IL-6) (full data available for 46 patients) were obtained. As shown in **Table 2**, in comparison to the non-critically ill patients, patients with critical illness had higher white blood cell and neutrophil counts, as well as higher levels of aspartate transaminase, blood urea nitrogen, creatine, D-dimer, creatine kinase, B-type natriuretic peptide, C-reactive protein, procalcitonin, and interleukin-6. Besides, patients with severe pneumonia had more severe hypoalbuminemia than patients exhibiting less critical forms of the disease.

**Table 2:** Comparison of laboratory findings, treatment measures and clinical outcomes between critically and non-critically ill COVID-19 pneumonia patient groups.

Characteristic	Normal range	Non-Critically ill (N=41)	Critically ill(N=22)	P-value
**Laboratory findings**				
Leukocyte (10^9^/L)	3.5–9.5	5·1 (4·3–7.2)	9·5 (7·8–12·4)	0·001
Lymphocyte (10^9/^L)	1.1–3.2	0·9 (0·72–1·17)	0·67 (0·44–1·07)	0·059
Neutrophil (10^9^/L)	1.80–6.3	3·69 (2·81–5·45)	8·06 (5·13–9·84)	0·002
Platelet (10^9^/L)	125–350	219 (168–296)	196 (155–231)	0·056
Total bilirubin, mmol/L	0–20	11 (8–15)	12·5 (8–17)	0·406
Alanine aminotransferase, U/L	9–50	24 (20–42)	38·5 (21–69)	0·217
Aspartate transaminase, U/L	15–40	28 (23–38)	50 (35–83)	0·001
Albumin, (mg/dL)	40.0–55.0	34·4 ± 4·6	30·8 ± 3·7	0·002
Blood urea nitrogen, mmol/L	2.8–7.2	4·7 (3·4–5·9	5·5 (4·9–6·9)	0·014
Creatinine, μmol/L	44–97	60 (55–67)	68·5 (58–83)	0·030
Glucose, mmol/L	3.9–6.1	7·4 (5·7–10·7)	9·3 (8–9·6)	0·121
Prothrombin time	11.5–14.6	15·9 (15·3–16·8)	16·2 (15·7–16·8)	0·829
Fibrinogen (N=57), g/L	2.00–4.00	5·35 (4·65–6·35) (N=38)	6·05 (5·16–6·84) (N=19)	0·101
D-dimer (N=58), mg/L	0.00–0.50	0·68 (0·48–0·98) (N=36)	1·14 (0·68–1·47) (N=22)	0·009
Creatine kinase, U/L	0.00–4.87	69 (52–90)	147·5 (64–283)	0·014
B-type natriuretic peptide (N=62), pg/ml	0.00–125.0	18·5 (10–53·5) (N=40)	64·5 (19–152) (N=22)	0·002
C-reactive protein, mg/L	0.0–6.0	20·4 (14·9–47·6)	54 (24·1–90)	0·021
Procalcitonin, ng/mL	0-0.5	0·06 (0·04–0·08)	0·12 (0·06–0·20)	0·001
Interleukin-6 (N=46), pg/ml	<3	8·4 (4·0–32·2) (N=30)	76·1 (32·2–103·1) (N=16)	<0·001
Treatment*				
High flow nasal cannula, N (%)		0	18 (81·8)	<0·001
Mechanical ventilation, N (%)		0	16 (72·7)	<0·001
Non-invasive, N (%)		0	14 (63·6)	<0·001
invasive, N (%)		0	10 (45·5)	<0·001
Extracorporeal membrane oxygenation, N (%)		0	6 (27·3)	<0·001
Antibiotics, N (%)		31 (75·6)	21 (95·5)	0·079
Antiviral, N (%)				
Kaletra, N (%)		33 (80·5)	19 (86·4)	0·733
Arbidol, N (%)		39 (95·1)	21 (95·5)	1·000
methylprednisolone, N (%)		8 (19·5)	19 (86·4)	0·001
thymosin alpha 1, N (%)		10 (24·4)	16 (72·7)	<0·001
Intravenous immunoglobulin, N (%)		8 (19·5)	13 (59·1)	<0·001
blood plasma transfusion, N (%)		1 (2·4)	11 (50·0)	<0·001
Intravenous albumin, N (%)		22 (53·7)	21 (95·5)	0·001
**Outcomes**				
Hospitalization, N (%)		0 (0)	5 (22·7)	0·004
Discharge, N (%)		41 (100)	15 (68·2)	<0·001
Hospital days, N (%)		17·2 ± 6·7	24·1 ± 5·5	<0·001
Death, N (%)		0 (0)	1 (4.5)	0·118

*Patients may receive more than one treatment item.

**Figure 2:**
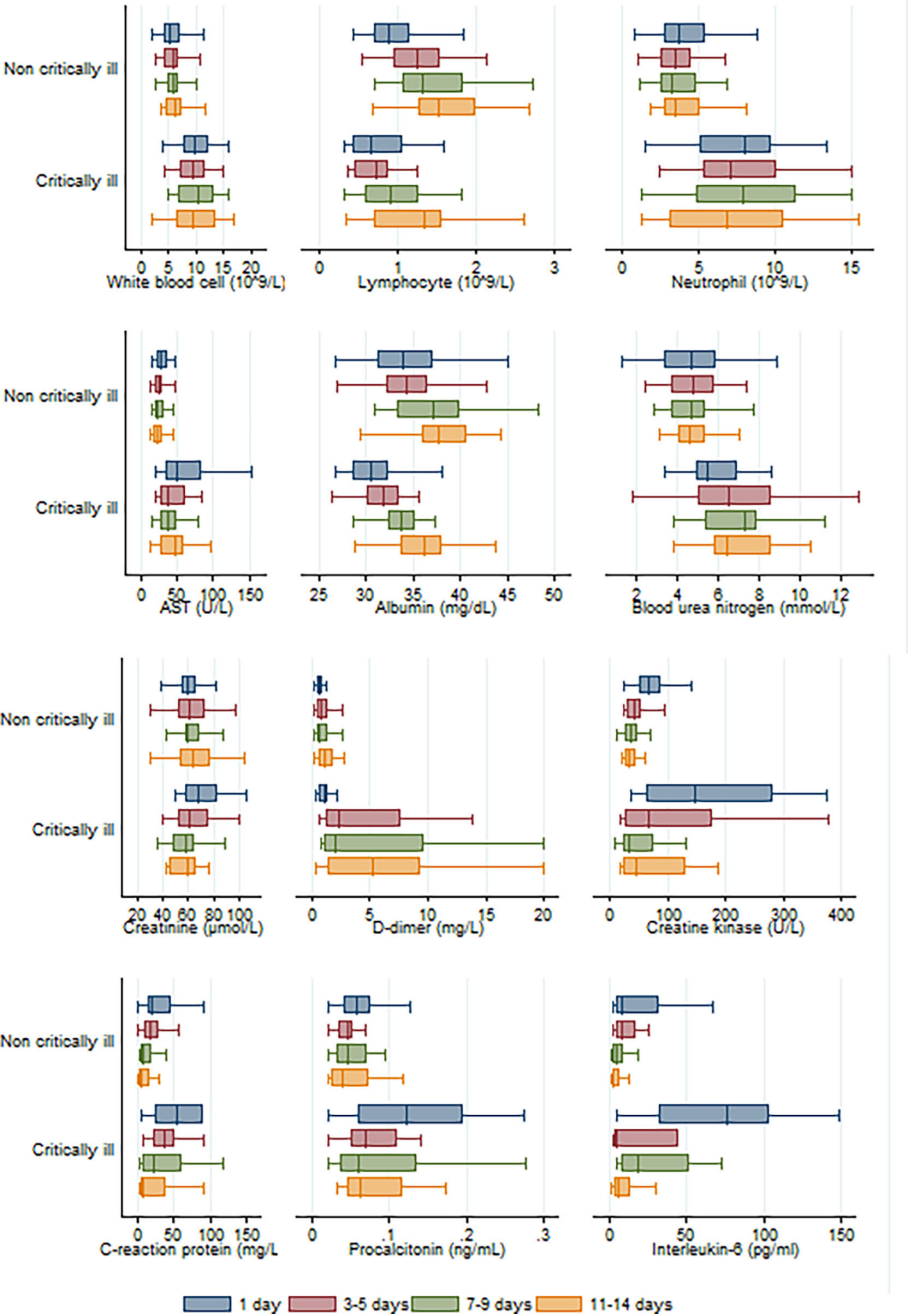
Longitudinal analysis of twelve laboratory parameters altered during the hospitalization in critically and non-critically ill patients.

As shown in **Figure 2**, longitudinal data revealed that statistical difference was still significant between COVID-19 pneumonia patient groups (critically ill *vs* non-critically ill) in terms of counts or levels of white blood cells, neutrophils, aspartate transaminase, blood urea nitrogen, and D-dimer within 3–5 days, 7–9 days, and 11–14 days of hospitalization. There was a significant statistical difference recorded for albumin and B-type natriuretic peptide levels between both groups within 3–5 or 7–9 days of hospitalization, but not within 11–14 days of hospitalization. A significant statistical difference was also observed for the interleukin-6 levels between the two groups within 7–9 days and 11–14 days of hospitalization. Compared to patients not critically ill with pneumonia, critically ill patients had lower lymphocyte counts within 3–5 days, 7–9 days, and 11–14 days of hospitalization. And the lymphocyte numbers gradually increased with the duration of hospitalization in pneumonia patients irrespective of disease severity.

### Treatment and Outcomes

A high flow nasal cannula was initially used in the treatment of 18 patients, of which 6 belonged to the critically ill group and strictly required a high flow nasal cannula, while the rest (12 patients) were switched to mechanical ventilation or extracorporeal membrane oxygenation (ECMO) (**Table 2** and **Figure 1**). Ultimately, mechanical ventilation was performed in 16 patients, 6 of whom received ECMO as rescue therapy (**Figure 1**).

A total of 52 (82·5%) patients received empirical antibiotic therapy (moxifloxacin, 33 [52·4%]; tazocin, 18 [18·6%]; ceftazidime, 6 [9·5%]; and Piperacillin, 6 [9·5%]) and antiviral therapy (Kaletra, 52 [82·5%] and Arbidol, 60 [95·2%]). Twenty-seven (42·9%) patients were given systematic methylprednisolone, while 26 (41·3%) patients received thymosin alpha 1. Additionally, intravenous immunoglobulin, blood plasma transfusion, and intravenous albumin were administered in 21 (33·3%), 12 (19·1%), and 43 (68·3%) patients, respectively. When compared to the non-critically ill group of patients, critically ill patients were given a higher dose of methylprednisolone, thymosin alpha 1, intravenous immunoglobulin, blood plasma transfusion, and intravenous albumin therapy (**Table 2**).

As of March 12, 2020, six critically ill patients were still hospitalized. Among these, four had been transferred to the general wards because of improved condition, while the remaining two patients, who are 79 and 92 years old, respectively, and are both supported by ECMO, are still in the ICU. A total of 56 patients (88.9%) have been discharged, and one patient (1·6%) died. The patient who died was 79 years old and underwent ECMO therapy before death. When compared with the non-critically ill patient group, critically ill patients had a lower discharge rate (68·2% *vs*. 100%, *p*<0·001), longer hospital stays (24·1 *vs*. 17·2 days, *p*<0·001), and a trend towards higher mortality (4·5% *vs*. 0, *p*=0·349) (**Table 2**).

### Interleukin-6 in Predicting the Severity of COVID-19 Pneumonia

**Figure 3:**
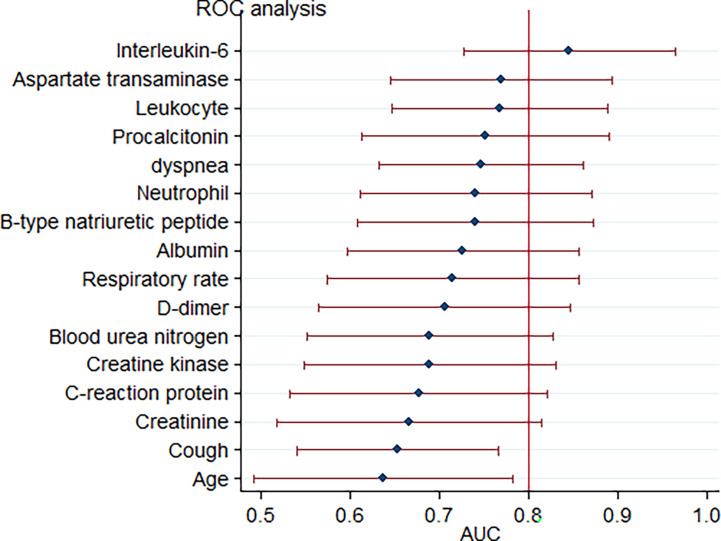
Forest plot for accuracy of various markers in predicting critical COVID-19-related pneumonia. Each marker is plotted as an area under the curve of the receiver operating characteristic curve (AUC) with a 95% confidence interval.

**Figure 4:**
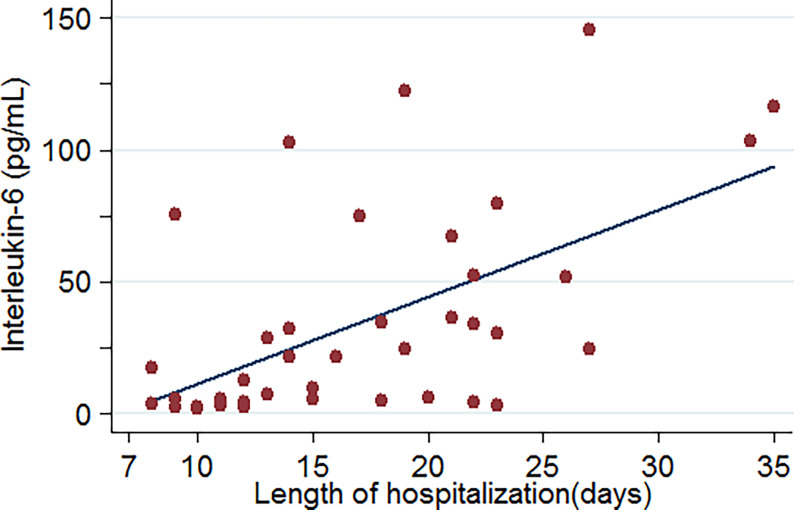
Relationship between IL-6 levels and length of hospitalization of COVID-19-related pneumonia patients (data were available for 46 patients).

Upon admission, the parameters that reached a statistically significant difference between patients with and without critical illness include age, presence of cough and dyspnoea, respiratory rate, white blood cell counts, neutrophil counts, and levels of aspartate transaminase, albumin, blood urea nitrogen, creatine, D-dimer, creatine kinase, B-type natriuretic peptide, C-reactive protein, procalcitonin, and interleukin-6. These were evaluated as potential predictors of severe COVID-19 pneumonia. Based on the ROC curve analysis, among single predictors, interleukin-6 has the highest AUC (0·85), indicative of excellent diagnostic accuracy (**Figure 3**). Further analysis revealed that there was a positive correlation between IL-6 levels and duration of hospital stay (R=0·58, *p*=0·0001) (**Figure 4**).

It was also inferred from the ROC curve analysis that the optimum cut-off value for interleukin-6 was 77·5 pg/mol. The sensitivity, specificity, positive likelihood ratio, negative likelihood ratio, positive predictive value, negative predictive value, and diagnostic accuracy were 50, 96·7, 15, 0·52, 88·9, 78·4, and 80·4%, respectively.

**Figure 5:**
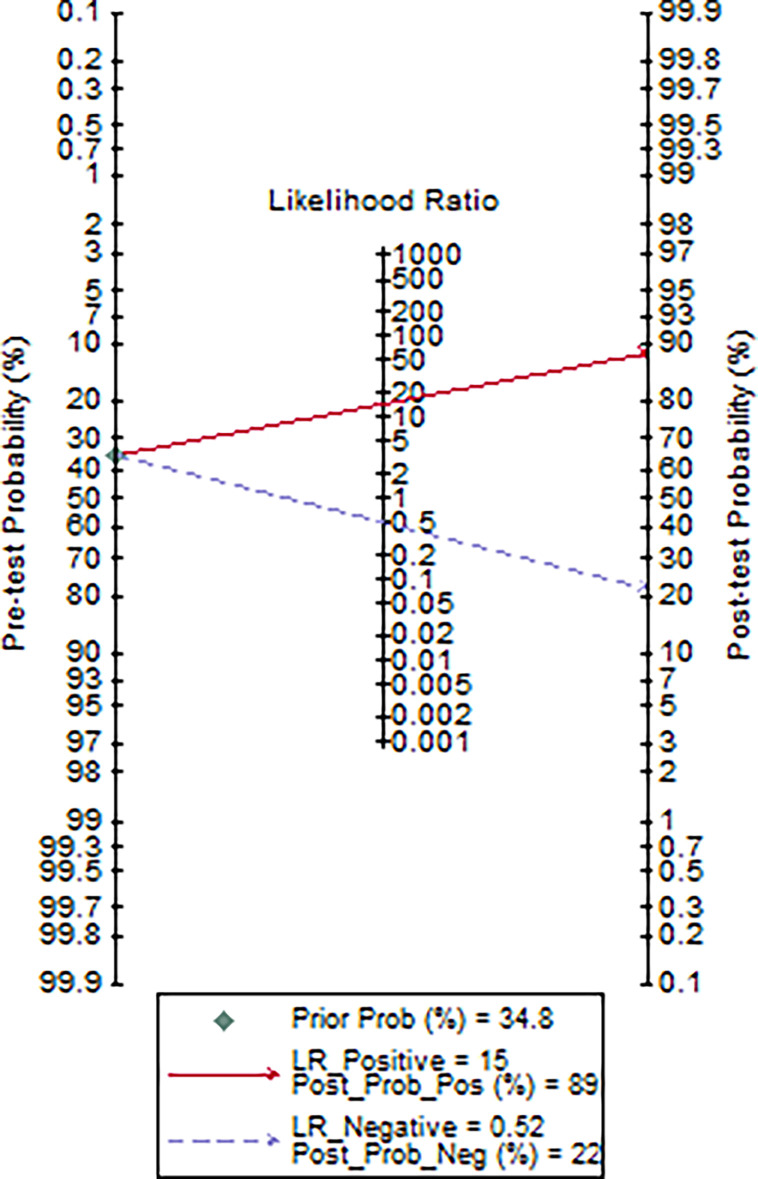
Fagan plot of IL-6 for the hospital stay prediction of critically ill COVID-19 patients (data were available for 46 patients).

Using a cut-off value of 77·5 and the incidence of severe pneumonia (34·8% in this study) as the pre-test probability, the resulting Fagan plot (**Figure 5**) shows that interleukin-6 levels can be clinically informative, as it increases the probability of the patient being classified into the hypoxemia group by up to 89% when positive, and lowers the probability by up to 22% when negative.

## Discussion

The earliest patient was an imported case who had a history of travel from Wuhan City, and the latest patient had an occult epidemic history (**Supplementary Figure 1**). A total of 28 (38·1%) patients were imported cases that had immediate travel history from Wuhan city. Fourteen (22·2%) patients had a history of direct exposure to confirmed patients. The rapid human-to-human transmission among close contacts is an important feature of COVID-19 infection (Wang et al., 2020). Early restriction of patient contact may reduce transmission of the disease. If individuals come into contact with confirmed patients with a history of travel to epidemic areas, such as Wuhan, they would be quarantined at designated hospitals for at least two weeks. Twenty-four (39·7%) patients had an occult epidemic history. This highlights the importance of not neglecting the possibility of transmission from asymptomatic patients, since most (80%) patients with COVID-19 infection would be mild and asymptomatic (Tian et al., 2020).

On average, critically ill patients were shown to be older than non-critically ill patients (61·5 *vs*. 52·9 years old, *p*<0·034) (**Table 1**). As of March 12, 2020, one patient died, while two were still alive but critically ill (**Figure 1**). The patient who died was 79 years old and underwent ECMO therapy before death. Zhou et al. (2020) showed that older age is one of the risk factors for mortality in adult inpatients with COVID-19 in Wuhan. In our cohort, fever was the most common symptom at the onset of the illness in patients with COVID-19 pneumonia (**Table 1**) occurring in 62 patients (98·4%), followed by cough (61·94%) and sputum (34·9%), which is in accordance with previous studies (Guan et al., 2020; Wang et al., 2020). The remaining patient was asymptomatic and had no fever from the moment of admission until the time he was discharged. He was hospitalized because he had a history of direct exposure to confirmed patients and evidence of pneumonia based on chest CT images. In addition, 16 (25·4%) patients had normal body temperatures at admission. These findings suggest that a normal fever scan cannot accurately diagnose COVID-19 pneumonia. An RT-PCR test and chest CT images may be useful in identifying COVID-19 pneumonia early and accurately in individuals that have an exposure history, although it is not known whether these measures are cost-effective or not. It also implies that measuring only the body temperature may not successfully identify all patients among individuals in public places such as airports, hospitals, and schools; 52·4% of patients had a temperature of <37·5°C, and only 1·6% of patients had a high temperature, i.e. >39·0°C (**Table 2**). These findings are consistent with a previous report by Guan et al. (2020). When compared with the non-critically ill patients, the patient group exhibiting critical illness had a higher incidence of cough and dyspnoea and a higher respiratory rate. These symptoms could be partly explained by histological findings from COVID-2019 pneumonia patients, in which lung tissue displayed pulmonary edema with hyaline membrane formation and desquamation of pneumocytes, indicating acute respiratory distress syndrome (Xu Z. et al., 2020).

Patients belonging to the critically ill group had higher white blood cell counts, neutrophil counts, and D-dimer levels than patients in the non-critically ill group. This indicated that severely ill patients get a stronger inflammatory response, as well as activation of coagulation induced by viral infection. Lymphocytopenia may be associated with cellular immune deficiency. Yang et al. suggested that the severity of lymphocytopenia may reflect the severity of COVID-2019 infection (Yang et al., 2020). Our study found that there was a trend for lower total lymphocytes in patients belonging to the critically ill group than those in the non-critically ill group, although it did not reach statistical significance upon admission (*p*=0·059). Longitudinal data indicated that when compared to non-critically ill COVID-19 pneumonia patients, critically ill patients had lower lymphocyte counts within 3–5 days, 7–9 days, and 11–14 days of hospitalization (**Figure 2**). Critically ill patients also had hypoalbuminemia and increased levels of aspartate transaminase, blood urea nitrogen, creatine, creatine kinase, B-type natriuretic peptide, which are more indicative of severe hepatic, kidney and myocardial injury (Wang et al., 2020). It has been shown that, similar to SARS-CoV, the COVID-19 virus exploits the angiotensin converting enzyme 2 (ACE2) receptor to gain entry into the cells (Baig et al., 2020). The most remarkable finding was the surface expression of ACE2 protein on lung alveolar epithelial cells and enterocytes of the small intestine (Hamming et al., 2004). Furthermore, ACE2 was present in arterial and venous endothelial cells and arterial smooth muscle cells in all tissues studied, including liver, kidney, cardiovascular, and brain tissues (Hamming et al., 2004).

Rapid production of IL-6 contributes to host defense during infection and tissue injury, but excessive IL-6 production causes severe inflammatory diseases (Kang et al., 2019). Our study showed that patients who were in critical condition had higher IL-6 levels than patients who were not (76·1 vs. 8·4 pg/mL, *p*<0·001) (**Table 2**). This means that severe COVID-19 infection could result in a cytokine storm. Our study showed that there was a good positive correlation between IL-6 levels and the length of hospitalization. Higher IL-6 levels were associated with longer hospital stays (R=0·58, *p*=0·0001) (**Figure 4**). Based on the ROC curve analysis, the AUC and the optimum cut-off value of interleukin-6 as a predictor of critical COVID-19 pneumonia cases were 0·85 and 77·5 pg/mol, respectively. The sensitivity, specificity, and diagnostic accuracy were 50, 96·7, and 80·4%, respectively. Using the incidence of critically ill COVID-19 pneumonia as the pre-test probability, the resulting Fagan plot shows that interleukin-6 can be clinically informative, as it increases the probability of a patient being classified into the hypoxemia group by up to 89% when positive and lowers the probability to 22% when negative (**Figure 5**). Longitudinal data also indicated a significant IL-6 decrease after hospitalization in both patients with and without critical stage COVID-19 pneumonia (**Figure 2**). There is evidence that IL-6 blockade strategy is beneficial for several inflammatory diseases, such as rheumatoid arthritis, juvenile idiopathic arthritis (Kang et al., 2019). It would be interesting to conduct a randomized clinical trial to test whether interfering with the IL-6 signalling axis in COVID-19 pneumonia patients is effective in treating the disease.

Yang et al. suggested that for non-critically ill patients, close follow-up is likely to be sufficient to manage the disease (Yang et al., 2020). Our study showed that patients with non-critical illness upon admission may advance to a critical stage later on. As shown in **Figure 1**, one COVID-19 pneumonia patient in the common type group and 13 patients in the severe type group advanced to the critically ill stage during hospitalization. This means that both critically ill and non-critically ill COVID-19 pneumonia patients need close monitoring and aggressive treatments.

Until now, no specific treatment has been recommended for coronavirus infections, except for meticulous supportive care (Wang et al., 2020). Mechanical ventilation is the main supportive treatment for critically ill patients (Yang et al., 2020). Three (50%) of the six patients who used ECMO recovered and got discharged. ECMO is an excellent candidate for refractory hypoxemia caused by severe acute respiratory distress syndrome, if it can be delivered in a medical center that has experience of using this form of therapy (Mi et al., 2018). Even without solid evidence for the effectiveness of antibacterial or antiviral therapies in the treatment of COVID-19 infections, 82·5% (52/63) of patients received antibacterial agents and nearly all the patients received antiviral therapy. For this study, we used Kaletra and Arbidol. A study on the Middle East Respiratory Syndrome Coronavirus (MERS-CoV) showed that the timing of the start of treatment with antiviral agents is important in most viral infections, and starting antiviral treatment early might lead to better outcomes (Momattin et al., 2013).

When compared with the non-critically ill patients in our study, the critically ill ones received higher levels of methylprednisolone (86·4% *vs*. 19·5%, *p*=0·001). The dose of methylprednisolone varied depending on disease severity (Wang et al., 2020). Systemic corticosteroids were shown to delay viral clearance in critically ill patients with MERS-CoV (Memish et al., 2020). However, a study on severe acute respiratory syndrome (SARS) suggested that the use of interferon alfacon-1 plus corticosteroids was associated with reduced disease-associated impaired oxygen saturation and more rapid resolution of radiographic lung abnormalities (Loutfy et al., 2003). Wu et al. reported that treatment with methylprednisolone may be beneficial for patients with COVID-19 pneumonia who develop acute respiratory distress syndrome (Wu et al., 2020).

When compared with the non-critically ill patients, critically ill ones had a lower discharge rate (68·2% *vs*. 100%, *p*<0·001) and longer hospital days (24·1 *vs*. 17·2 days, *p*<0·001), as well as a trend towards higher mortality (4·5% *vs.* 0, *p*=0·349). The mortality of critically ill patients in our study was significantly lower than that reported by Yang et al. in Wuhan (4·5% *vs*. 61·5%) (Yang et al., 2020). Early isolation, early diagnosis, and early management might have collectively contributed to the reduction in mortality (Guan et al., 2020). In addition, thymosin alpha 1, intravenous administration of immunoglobulin, blood plasma transfusion and intravenous albumin therapy may also improve the clinical outcome.

The limitation of this study is that it is a retrospective study from a single center, and the sample size was small. Patients had a variety of different treatments (for example: antivirals, antibiotics, intravenous immunoglobulin, blood plasma transfusion, and intravenous albumin). This made it very difficult to interpret any other outcome data objectively. Therefore, it would be interesting to conduct a randomized clinical trial to assess the effect of different therapeutic strategies for patients with or without COVID-19 pneumonia in future studies. Additionally, only patients with pneumonia were enrolled, so our results may be not applicable to patients without pneumonia.

## Conclusions

In conclusion, both critically ill and non-critically ill COVID-19 pneumonia patients need close monitoring and aggressive treatments. There was a statistically significant difference between critically ill and non-critically ill COVID-19 pneumonia patients in terms of age, some symptoms, laboratory findings, treatments, and outcomes. Interleukin-6 levels upon admission is a good predictor of the disease, and it is associated with the length of hospitalization.

## Data Availability Statement 

The raw data supporting the conclusions of this article will be made available by the authors, without undue reservation.

## Ethics Statement

This study protocol was approved by the Ethics Committee of the First Affiliated Hospital of Wenzhou Medical University. The committee decided to waive the need for written informed consent from the participants studied in this analysis as the data were analyzed retrospectively and anonymously.

## Author Contributions 

WH conceived the study and carried out the majority of the work. WH, QC, SQ, YW, and JP participated in data collection. WH conducted data analysis and drafted the manuscript. ZB, VZ, MZ, and JP helped to finalize the manuscript. All authors contributed to the article and approved the submitted version.

## Funding

This work was supported by Wenzhou Science and Technology Bureau (Number: Y2020010) and Wenzhou Key Technology Breakthrough Program on Prevention and Treatment for COVID-19 Epidemic, No. ZG2020012.

## Conflict of Interest

The authors declare that the research was conducted in the absence of any commercial or financial relationships that could be construed as a potential conflict of interest.

## Supplementary Material

Supplementary Figure 1 Bar graph depicting travel history of patients with COVID-19-related pneumonia.

Supplementary Figure 2 Representative computed tomography images from a patient with COVID-19-related pneumonia. **(A)** High-density local patches and masses are seen, and the edges are blurred and thickened in the left lung; **(B)** Ground glass opacities in two lungs.

## References

[B1] (2020) Notice on the Novel Coronavirus Infection Diagnosis and Treatment Plan (Trial Version Fifth). Available at: http://www.nhc.gov.cn/yzygj/s7653p/202002/3b09b894ac9b4204a79db5b8912d4440.shtml.

[B2] Baig A. M. Khaleeq A. Ali U. Syeda H. (2020). Evidence of the COVID-19 Virus Targeting the CNS: Tissue Distribution, Host-Virus Interaction, and Proposed Neurotropic Mechanisms. ACS Chem. Neurosci. 11 (7), 995–998. 10.1021/acschemneuro.0c00122 32167747

[B3] Guan W. J. Ni Z. Y. Hu Y. Liang W. H. Ou C. Q. He J. X.. (2020). Clinical Characteristics of Coronavirus Disease 2019 in China. N. Engl. J. Med. 382 (18), 1708–1720. 10.1056/NEJMoa2002032 32109013 PMC7092819

[B4] Hamming I. Timens W. Bulthuis M. L. Lely A. T. Navis G. Van Goor H. (2004). Tissue Distribution of ACE2 Protein, the Functional Receptor for SARS Coronavirus. A First Step in Understanding SARS Pathogenesis. J. Pathol. 203, 631–637. 10.1002/path.1570 15141377 PMC7167720

[B5] Hong W. Dong L. Huang Q. Wu W. Wu J. Wang Y. (2011). Prediction of Severe Acute Pancreatitis Using Classification and Regression Tree Analysis. Dig. Dis. Sci. 56, 3664–3671. 10.1007/s10620-011-1849-x 21833749

[B6] Hong W. Lin S. Zippi M. Geng W. Stock S. Basharat Z.. (2017). Serum Albumin Is Independently Associated With Persistent Organ Failure in Acute Pancreatitis. Can. J. Gastroenterol. Hepatol. 2017, 5297143. 10.1155/2017/5297143 29147647 PMC5632885

[B7] Hong W. Zimmer V. Basharat Z. Zippi M. Stock S. Geng W.. (2020). Association of Total Cholesterol With Severe Acute Pancreatitis: A U-Shaped Relationship. Clin. Nutr. 39, 250–257. 10.1016/j.clnu.2019.01.022 30772093

[B8] Kang S. Tanaka T. Narazaki M. Kishimoto T. (2019). Targeting Interleukin-6 Signaling in Clinic. Immunity 50, 1007–1023. 10.1016/j.immuni.2019.03.026 30995492

[B9] Kumar A. Zarychanski R. Pinto R. Cook D. J. Marshall J. Lacroix J.. (2009). Critically Ill Patients With 2009 Influenza A(H1N1) Infection in Canada. JAMA 302, 1872–1879. 10.1001/jama.2009.1496 19822627

[B10] Liao X. Wang B. Kang Y. (2020). Novel Coronavirus Infection During the 2019-2020 Epidemic: Preparing Intensive Care Units-the Experience in Sichuan Province, China. Intensive Care Med. 46, 357–360. 10.1007/s00134-020-05954-2 32025779 PMC7042184

[B11] Loutfy M. R. Blatt L. M. Siminovitch K. A. Ward S. Wolff B. Lho H.. (2003). Interferon Alfacon-1 Plus Corticosteroids in Severe Acute Respiratory Syndrome: A Preliminary Study. JAMA 290, 3222–3228. 10.1001/jama.290.24.3222 14693875

[B12] Memish Z. A. Perlman S. Van Kerkhove M. D. Zumla A. (2020). Middle East Respiratory Syndrome. Lancet 395 (10229), 1063–1077. 10.1016/S0140-6736(19)33221-0 32145185 PMC7155742

[B13] Mi M. Y. Matthay M. A. Morris A. H. (2018). Extracorporeal Membrane Oxygenation for Severe Acute Respiratory Distress Syndrome. N. Engl. J. Med. 379, 884–887. 10.1056/NEJMclde1804601 30157406

[B14] Momattin H. Mohammed K. Zumla A. Memish Z. A. Al-Tawfiq J. A. (2013). Therapeutic Options for Middle East Respiratory Syndrome Coronavirus (MERS-CoV)–possible Lessons From a Systematic Review of SARS-CoV Therapy. Int. J. Infect. Dis. 17, e792–e798. 10.1016/j.ijid.2013.07.002 23993766 PMC7110699

[B15] Peeri N. C. Shrestha N. Rahman M. S. Zaki R. Tan Z. Bibi S.. (2020). The SARS, MERS and Novel Coronavirus (COVID-19) Epidemics, the Newest and Biggest Global Health Threats: What Lessons Have We Learned? Int. J. Epidemiol. 49 (3), 717–726. 10.1093/ije/dyaa033 32086938 PMC7197734

[B16] Tian S. Hu N. Lou J. Chen K. Kang X. Xiang Z.. (2020). Characteristics of COVID-19 Infection in Beijing. J. Infect. 80 (4), 401–406. 10.1016/j.jinf.2020.02.018 32112886 PMC7102527

[B17] Wang D. Hu B. Hu C. Zhu F. Liu X. Zhang J.. (2020). Clinical Characteristics of 138 Hospitalized Patients With 2019 Novel Coronavirus-Infected Pneumonia in Wuhan, China. JAMA 323 (11), 1061–1069. 10.1001/jama.2020.1585 32031570 PMC7042881

[B18] Wu C. Chen X. Cai Y. Xia J. Zhou X. Xu S.. (2020). Risk Factors Associated With Acute Respiratory Distress Syndrome and Death in Patients With Coronavirus Disease 2019 Pneumonia in Wuhan, China. JAMA Intern. Med. 180 (7), 934–943. 10.1001/jamainternmed.2020.0994 32167524 PMC7070509

[B19] Xu Y. H. Dong J. H. An W. M. Lv X. Y. Yin X. P. Zhang J. Z.. (2020). Clinical and Computed Tomographic Imaging Features of Novel Coronavirus Pneumonia Caused by SARS-Cov-2. J. Infect. 80 (4), 394–400. 10.1016/j.jinf.2020.02.017 32109443 PMC7102535

[B20] Xu Z. Shi L. Wang Y. Zhang J. Huang L. Zhang C.. (2020). Pathological Findings of COVID-19 Associated With Acute Respiratory Distress Syndrome. Lancet Respir. Med. 8 (4), 420–422. 10.1016/S2213-2600(20)30076-X 32085846 PMC7164771

[B21] Yang X. Yu Y. Xu J. Shu H. Xia J. Liu H.. (2020). Clinical Course and Outcomes of Critically Ill Patients With SARS-CoV-2 Pneumonia in Wuhan, China: A Single-Centered, Retrospective, Observational Study. Lancet Respir. Med. 8 (5), 475–481. 10.1016/S2213-2600(20)30079-5 32105632 PMC7102538

[B22] Zhou F. Yu T. Du R. Fan G. Liu Y. Liu Z.. (2020). Clinical Course and Risk Factors for Mortality of Adult Inpatients With COVID-19 in Wuhan, China: A Retrospective Cohort Study. Lancet 395 (10229), 1054–1062. 10.1016/S0140-6736(20)30566-3 32171076 PMC7270627

