# Supplementary figures and images for: Critically Ill vs. Non-Critically Ill Patients With COVID-19 Pneumonia: Clinical Features, Laboratory Findings, and Prediction
Source: Front Cell Infect Microbiol. 2021 Jul 13;11:550456. doi: 10.3389/fcimb.2021.550456 (PMC8313893; doi:10.3389/fcimb.2021.550456)

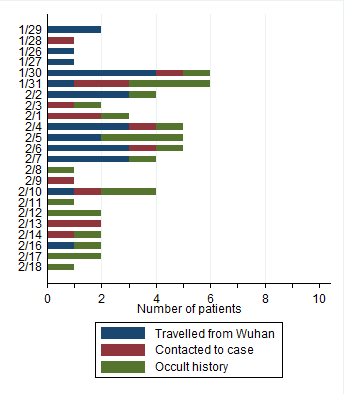

Supplement: Supplementary Figure 1 — Bar graph depicting travel history of patients with COVID-19-related pneumonia. [file Image_1.tif]

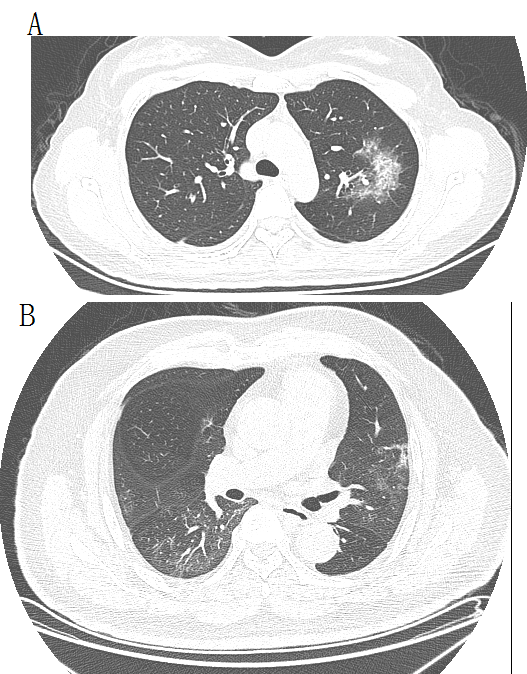

Supplement: Supplementary Figure 2 — Representative computed tomography images from a patient with COVID-19-related pneumonia. (A) High-density local patches and masses are seen, and the edges are blurred and thickened in the left lung; (B) Ground glass opacities in two lungs. [file Image_2.tif]
